# Synchronous Uterine Metastases from Breast Cancer: Case Study and Literature Review

**DOI:** 10.7759/cureus.1840

**Published:** 2017-11-13

**Authors:** Aisha Akhtar, Atul Ratra, Yana Puckett, Abu Baker Sheikh, Catherine A Ronaghan

**Affiliations:** 1 Department of Surgery, Texas Tech University Health Sciences Center; 2 Internal Medicine, Texas Tech University Health Sciences Center; 3 Student, Texas Tech University Health Sciences Center

**Keywords:** breast cancer, endometrial cancer, metastasis, extra-genital

## Abstract

Breast cancer rarely metastasizes to the uterus. Here, we report two breast cancer patients with synchronous metastases to the uterus. Case 1 highlights a 46-year-old female with invasive ductal carcinoma who presented with a breast mass and was found to have uterine enlargement on positron emission tomography (PET) scan. Biopsy revealed a metastatic 4 mm focus of breast cancer in the background of endometrial hyperplasia. Case 2 reports a 62-year-old postmenopausal female diagnosed with lobular carcinoma of the breast following an abnormal screening mammogram. A routine pap smear necessitated further workup, revealing simultaneous endometrial and cervical metastasis. Both patients did not have any gynecologic symptoms and presented a diagnostic challenge.

## Introduction and background

Breast cancer is the most common cancer worldwide and accounts for 29% of all cancers in women. It is the second leading cause of cancer mortality among women and the leading cause of cancer mortality among women 29-60 years of age [1]. Invasive ductal carcinoma (IDC) of the breast and invasive lobular carcinoma (ILC) of the breast account for 75% and 15% of all cases of breast cancer, respectively [2].

Six percent of breast cancer patients present with metastases and approximately 30% develop metastases following definitive treatment [3-4]. ILC is more likely to have metastases at presentation than IDC [5]. Although the most common sites of breast cancer metastasis, lung/pleura, liver, bone, and brain, are similar in ductal as well as lobular carcinoma, lobular carcinoma is more likely to metastasize to the gastrointestinal system, gynecologic organs, peritoneum-retroperitoneum, adrenal glands, and bone marrow [6].

Metastases to genital organs from extra-genital cancer sites are extremely rare. When present, ovaries are the most common site of metastasis due to peritoneal spread, accounting for 75.8% of metastases to genital organs [7-8]. Metastases to genital organs other than the ovaries thus pose a significant diagnostic challenge. Here, we are presenting two cases of breast cancer with metastases to the endometrium.

## Review

Case 1

A 42-year-old Hispanic woman with no known comorbidities presented to the emergency room with a two-month history of a painful and enlarging right breast mass, extending to the right axilla. Her review of systems was positive for anorexia and an unspecified amount of weight loss over the last two months. Social history was negative for smoking, alcohol, or illicit drug use. Family history was negative for any cancer. Physical examination revealed a firm, tender, and immobile mass in the right breast at the one o’ clock position, concerning for breast cancer. An abdominal exam revealed an enlarged uterus approaching the xiphoid process. An ultrasound examination revealed a mass at the 12 o’ clock position in the right breast, associated with a skin thickening of 4.2 mm and axillary lymphadenopathy, the largest measuring 1.2 cm in the long axis. There were two, right supraclavicular nodes measuring 8.5 mm and 9.7 mm, respectively. A core needle biopsy of breast mass revealed a poorly differentiated IDC with modified Scarf-Bloom-Richardson grade III. An immunohistochemical (IHC) analysis demonstrated that the tumor was moderately positive for estrogen receptor (ER) at 15%, strongly positive for progesterone receptor (PR) at 50%, Ki-67 unfavorable at 30%, and HER2/NEU-positive by fluorescence in situ hybridization (FISH). The axillary lymph node biopsy was also consistent with metastatic breast cancer.

**Figure 1:**
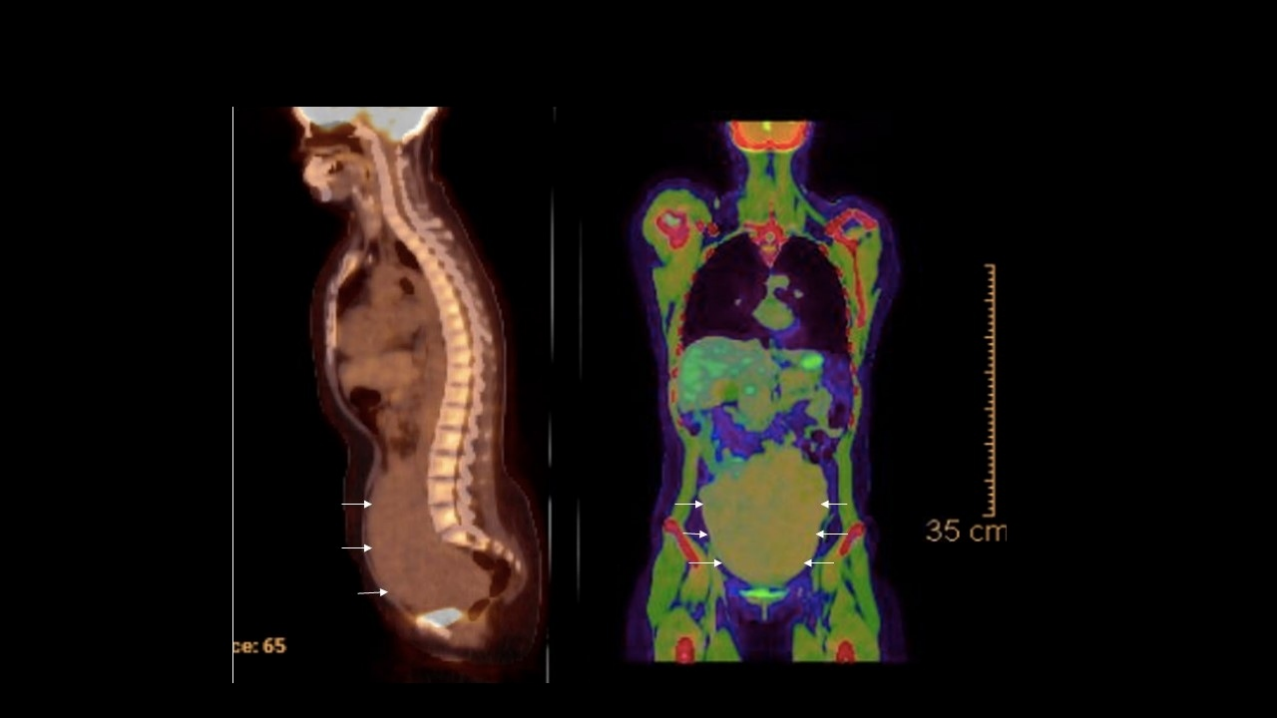
PET CT of patient shows a large mass in the pelvis (white arrows) PET: positron emission tomography; CT: computed tomography

A magnetic resonance imaging (MRI) breast was obtained to rule out any malignancy in the contralateral breast, which only demonstrated a 5.5 cm right breast mass (clinical stage T4N2). The staging workup, including the bone scan and computed tomography (CT) scan of the chest, were unremarkable. However, a positron emission tomography (PET) scan demonstrated massive uterine enlargement with diffuse hypermetabolic activity and bilateral hydronephrosis due to external compression (Figure 1).

A gynecology service was consulted, which performed an endometrial biopsy. The histopathology revealed a stromal adenocarcinoma, positive for GATA-3, pancytokeratin, and ER on IHC, suggesting breast cancer metastasis. The endometrial biopsy was morphologically similar to the specimen obtained from breast biopsy, affirming endometrial metastasis of breast cancer.

The patient was discussed during a multidisciplinary conference and recommended to undergo systemic chemotherapy followed by surgery. She completed six cycles of docetaxel, carboplatin, trastuzumab, and pertuzumab. She had a good clinical response with a barely palpable, vague right breast mass and a decrease in uterine size to the level of the umbilicus. Restaging the PET scan showed no hypermetabolic activity. She then underwent a total abdominal hysterectomy and a bilateral salpingo-oophorectomy. Final pathology showed a small metastatic deposit to the left ovary, but no evidence of residual metastases in the endometrium. Given the stage IV disease at presentation, the patient was started on maintenance chemotherapy with trastuzumab and pertuzumab. She continues to be on maintenance therapy for over one year without any evidence of recurrence or progression.

Case 2

**Figure 2:**
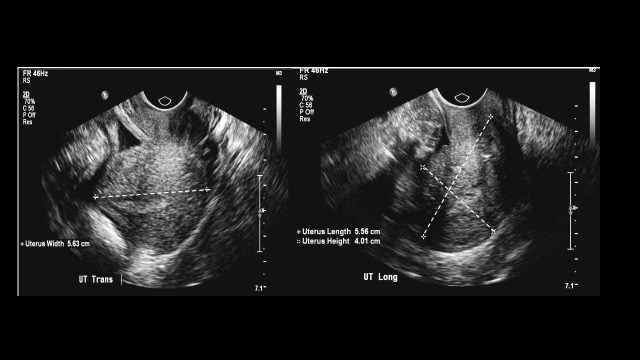
Ultrasound of the abdomen shows a large intrauterine mass.

A 62-year-old Caucasian woman with a long-standing history of smoking presented after a screening mammogram revealed a suspicious 14 mm mass in the superior aspect of the left breast. Her previous mammogram was five years ago, which was normal. Family history was positive for breast cancer in mother and sister. A physical examination revealed a 1.5 cm, palpable, immobile, nontender mass at the 12 o’clock position in the left breast. An ultrasound showed a 2.9 x 2.5 x 2.0 cm mass at the 12 o’ clock position of the left breast and right axillary lymphadenopathy. Provided contralateral axillary lymphadenopathy, an MRI was obtained to look for an occult right breast lesion, which showed an axillary tail mass of 1 x 2 cm with suspicious lymph nodes in the left breast. A core needle biopsy of the left breast mass was consistent with invasive lobular carcinoma along with fragments of a sclerosing papilloma. IHC staining demonstrated that the tumor specimen was strongly positive for ER and PR at 90% and 50%, respectively, Ki-67 of 5%, and HER2/NEU negative by FISH. A staging CT scan of chest, abdomen, and pelvis revealed a mass in the left breast as well as a right axillary tail mass and an enlarged uterus. A biopsy of the right axillary tail mass and lymph node also showed findings similar to those from the left breast mass. A routine pap smear necessitated further workup, revealing simultaneous endometrial and cervical metastasis. Given the enlarged uterus of 5.6 x 4.0 cm with an endometrium of 5 mm, a gynecology service was consulted and an endometrial biopsy was obtained, demonstrating metastatic lobular carcinoma, also involving the endocervix. This has been presented in Figure 2.

**Figure 3:**
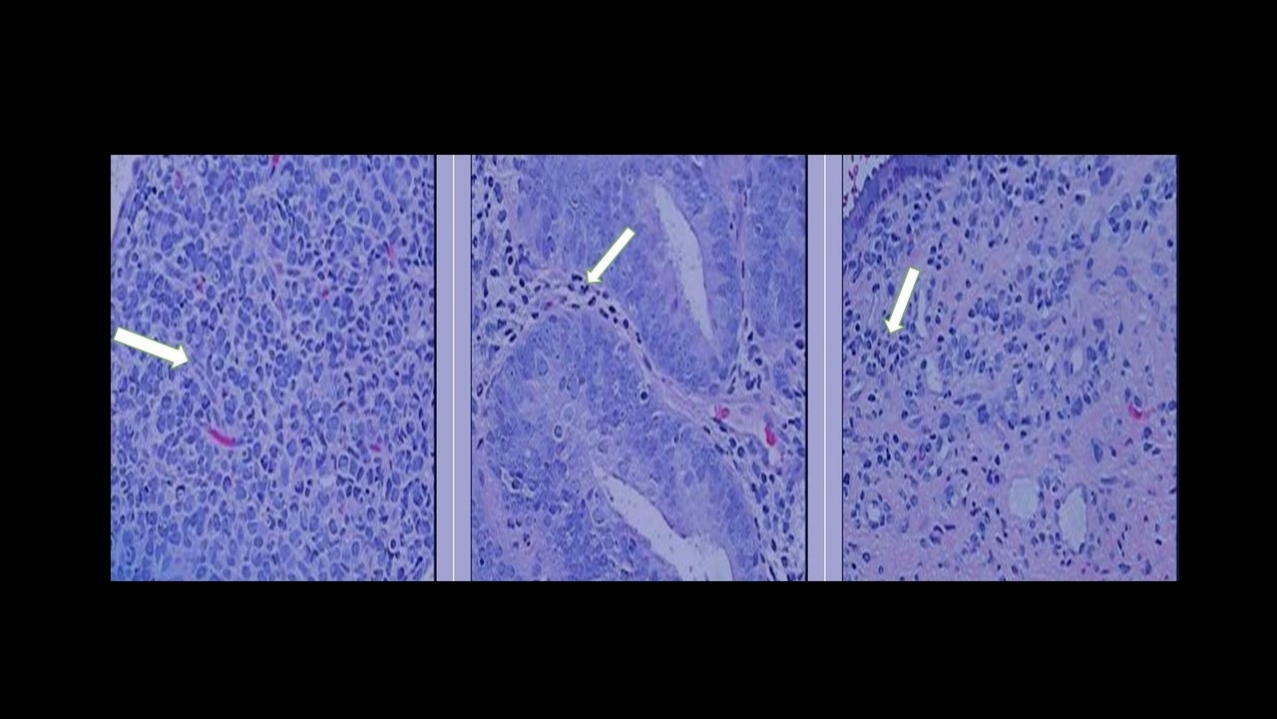
Endometrial biopsy revealed a tumor specimen with morphological features compatible with metastatic lobular carcinoma (arrows).

IHC staining of the endometrial biopsy specimen was positive for pancytokeratin, vimentin, ER, PR, and GATA-3 and negative for PAX-8. This has been presented in Figure 3.

The patient was discussed during a multidisciplinary conference, and the consensus was that the patient has a bilateral breast lobular carcinoma with metastases of the right breast carcinoma to the right axillary lymph nodes as well as the endometrium. She was offered hormonal and systemic chemotherapy with or without hysterectomy. The patient declined any further treatment and was lost to follow-up.

Discussion

We have described two cases of breast cancer with metastases to the uterus. Case 1 was a patient with IDC who presented with endometrial metastases. Case 2 was a patient with ILC who presented with endometrial as well as endocervical metastases. These cases are unique due to their unusual pattern of metastases involving only the endometrial lining and/or cervix.

Metastasis to genital organs other than ovaries from extra-genital cancers is extremely uncommon. Mazur et al. studied 325 patients with metastases to genital organs from all nonhematologic cancers, and only identified seven extra-genital tumors with endometrial metastases [8]. Only two of these had uterine metastases from breast cancer.

Kumar et al. studied 63 cases of uterine metastases from a variety of extra-genital cancers, primarily based on an autopsy examination [7]. Breast cancer accounted for approximately 43% of these cases, followed by colon cancer (18%), stomach cancer (11%), and pancreatic cancer (11%). Although metastases primarily involved the myometrium only (64%), 33% of patients also had endometrial involvement. Only two patients had isolated endometrial involvement, both of whom were detected on autopsy examination. This study suggests that uterine metastases from extra-genital cancers occur in the context of disseminated disease. These metastases usually stay overt given the short life expectancy thereafter. However, both patients presented here had synchronous isolated uterine metastases from breast cancer. Isolated uterine metastases at diagnosis presented a significant therapeutic challenge to us. Although the disease was considered to be stage IV, surgical treatment of isolated metastases was considered in both patients. Such an approach would definitely be controversial based on current guidelines. However, one patient who underwent surgery is clinically disease-free until recent follow-up.

We conducted a literature search of all patients with uterine metastases of breast cancer, utilizing PUBMED. We identified a total of 23 previously published cases, which are summarized in Table 1, along with the two cases we have reported [9-23].

**Table 1:** Literature review of all cases of breast cancer with uterine metastases utilizing PUBMED ER: estrogen receptor; PR: progesterone receptor; Her2: human epidermal growth factor receptor 2; TVUS: transvaginal ultrasound; CA15-3: cancer antigen 15-3; CEA: carcinoembryonic antigen

	Publication	Age	Histology	ER, PR, Her2	Stage at Dx	Met Sites	Synch	Presentation/Symptoms
1	Karvouni [9]	51	Ductal	ER+, PR-	TxN1M0	Endometrium, Cervix, Liver, Bone	No	Vaginal bleed
2	Hara [10]	44	Lobular	ER+, PR-, Her2-	T3aN1M0	Endometrium	No	Vaginal bleed
3	Arslan [11]	57	Ductal	ER+, PR+, Her2-	T1bN3aM0	Endometrium, Myometrium	No	Abd pain, distention
4	Erkanli [12]	63	Mixed	ER+, PR+, Her2+	T2N1M0	Endometrium	No	No symptoms, abnormal TVUS, elevated CA15-3
5	Scopa [13]	50	Lobular	ER-, PR+, Her2+	TxN3M0	Endometrium, Myometrium, Cervix, Ovaries, Fallopian tubes	No	Vaginal bleed
6	Scopa [13]	81	Lobular	ER+, PR+	T1N3M0	Endometrium, Myometrium, Cervix	No	Vaginal bleed
7	Sinkre [14]	58	Metaplastic	ER-, PR+, Her2-	T2N0M0	Endometrium, Myometrium, Ovary	No	Vaginal bleed
8	Giordano [15]	72	Lobular	ER+, PR+	T2N1M0	Endometrium, Myometrium, Cervix, Ovary	No	Vaginal bleed
9	Giordano [15]	77	Lobular	N/A	T2N1M0	Endometrium	No	Vaginal bleed
10	Kennebeck [16]	71	Ductal	ER-, PR-, Her2-	T1N1M0	Endometrium, Cervix, Vagina	No	No symptoms, elevated CEA
11	Al-Brahim [17]	53	Lobular	ER+, PR-	T2N1M0	Endometrium	No	Endometrial polyp, vaginal bleed
12	Ramalingam [18]	59	Ductal	ER+, PR+	IIIA	Endometrium, Urinary bladder	No	Urinary frequency, pelvic mass
13	Huo [19]	66	Ductal	ER-, PR-, Her2-	T2N0M0	Endometrium, Myometrium	No	No symptoms, elevated CEA, abnormal TVUS
14	Binstock [20]	43	Ductal	ER+, PR+, Her2 -	IIA	Endometrium, Myometrium, Fallopian tubes, Ovaries, Cervix, Bones	No	Vaginal bleed
15	Toyoshima [21]	62	Lobular	ER+, PR+, Her2+	T2N1M0	Fibroids, Myometrium	No	Abdominal compression, fibroids, elevated CEA, and CA15-3
16	Bezpalko [22]	47	Lobular	ER+, PR+, Her2-	IV	Endometrium, Bone, Bone marrow, Gallbladder	Yes	Vaginal bleed, breast edema and induration, cholecystitis
17	Houghton [23]	62	Lobular	N/A	T2N1M0	Endometrium	No	Endometrial polyp
18	Houghton [23]	92	Lobular	N/A	Localized	Endometrium	No	Vaginal bleed, endometrial polyp
19	Corley [24]	58	Ductal	N/A	N/A	Endometrium, Pleura, Peritoneum, Ovaries	No	Endometrial polyp, vaginal bleed
20	Alvarez [25]	69	Lobular	ER+, PR+	T2N1M0	Endometrium, Bone	No	Endometrial polyp, vaginal bleed,
21	Lambot [26]	70	Apocrine	ER+	LN+ T1cN1M0	Endometrium		Endometrial polyp, vaginal bleed
22	Sullivan [27]	83	Ductal	ER+, PR-	LN+	Endometrium	No	Uterine enlargement, endometrial polyp
23	Aranda [28]		Lobular	N/A	Localized	Endometrium	No	Endometrial polyp
24	Our case # 1	42	Ductal	ER+, PR+, Her2-	IV	Endometrium	Yes	Breast mass
25	Our case # 2	62	Lobular	ER+, PR+	IV	Endometrium	Yes	Breast mass

Out of 23, nine were ductal and 13 were lobular. The remaining three were mixed, apocrine, and metaplastic. Fifteen were ER positive, 13 were PR positive, and eight cases were Her2-negative. Only one case was reported with the synchronous presentation of metastatic breast cancer with endometrial metastasis. Vaginal bleeding was the most common presenting symptom. Only one patient presented with uterine enlargement.

A majority of patients with uterine metastases were positive for hormone receptors. It is unclear whether there is a relation between hormone positive status and risk of uterine metastases. We know tamoxifen, commonly used for hormone receptor-positive breast cancer, leads to endometrial hyperplasia. It is plausible that these endometrial changes and the associated angiogenesis lead to a favorable microenvironment for the seeding of breast cancer metastases.

The uterine metastases of extra-genital cancer can present a diagnostic challenge for physicians as well as pathologists. Even when appropriate workup is done, it may be difficult to distinguish between uterine cancer and the uterine metastases of breast cancer. Both have a glandular architecture on histopathology and both are likely to demonstrate positivity for hormone receptors. Immunohistochemical stains like GATA3 and cytokeratin should be employed in such cases, which have a sensitivity of 86% for breast cancer [29].

## Conclusions

In conclusion, synchronous endometrial metastases from breast cancer are extremely rare and can pose a significant diagnostic and therapeutic challenge. In the setting of isolated uterine metastases from breast cancer, surgery might be a viable option, especially if symptoms are present. However, it is unknown if surgery in these cases would prolong survival. The long-term follow-up of such patients is warranted.
